# Phenolic Compounds from Leaves and Flowers of *Hibiscus roseus*: Potential Skin Cosmetic Applications of an Under-Investigated Species

**DOI:** 10.3390/plants10030522

**Published:** 2021-03-10

**Authors:** Luana Beatriz dos Santos Nascimento, Antonella Gori, Andrea Raffaelli, Francesco Ferrini, Cecilia Brunetti

**Affiliations:** 1 Department of Agri-Food Production and Environmental Sciences (Florence), University of Florence, Sesto Fiorentino, 50019 Florence, Italy; luanabeatriz.dossantosnascimento@unifi.it (L.B.d.S.N.); francesco.ferrini@unifi.it (F.F.); 2 National Research Council of Italy, Institute for Sustainable Plant Protection (IPSP), Sesto Fiorentino, 50019 Florence, Italy; 3 Institute of Life Sciences—S. Anna School of Advanced Studies, 56127 Pisa, Italy; andrea1.raffaelli@santannapisa.it; 4 VALUE Laboratory on Green, Health & Wellbeing, University of Florence, 50019 Florence, Italy

**Keywords:** anti-collagenase, antioxidant, flavonoids, flowers, herbal cosmetics, hydroxycinnamic acids, LC-MS/MS-MRM, leaves, skin-care, sun protection

## Abstract

The use of plant extracts in skin-care cosmetics is a modern trend due to their richness in polyphenols that act as anti-aging molecules. *Hibiscus roseus* is a perennial species naturalized in Italy, with beautiful soft pink flowers; its phenolic composition and biological activities have not been studied yet. The aim of this study was to characterize and quantify the phenolics and to evaluate the antioxidant, sun protection factor (SPF), and anti-collagenase activities of the ethanolic extracts of *H. roseus* leaves (HL) and flowers (HF). *p*-Coumaric, chlorogenic, and *trans*-ferulic acids derivatives as well as quercetin and kaempferol flavonoids were the main phenolic compounds detected. Catechin, epicatechin, kaempferol-3-*O*-rutinoside, kaempferol-3-*O*-glucoside, kaempferol-7-*O*-glucoside, tiliroside, oenin, and peonidin-3-*O*-glucoside were detected only in HF, while phloridzin was exclusive from HL, which also showed greater amounts of hydroxycinnamic acid derivatives. HF was richer in flavonoids and total phenolics, also exhibiting greater antioxidant capacity. The SPF and anti-collagenase activity of both extracts were similar and comparable to those of synthetic standards. The overall results demonstrate that *H. roseus* extracts are promising sources of bioactive phenolic compounds that could be potentially applied as anti-aging agents in skin-care cosmetics.

## 1. Introduction

The use of cosmetics is ancient, and its history shapes in parallel with that of the humankind [1,2]. Skin-care cosmetics are some of the most important products, being the major category in this industry [1,3]. Therefore, the interest in skin-care has become widespread, triggering the demand for effective products derived from natural sources [2].

The recent awareness about the environment, healthcare, and the minor usage of synthetic chemicals led to an increasing interest in plant-based cosmetics, which now represent one-third of the entire cosmetic sector [1,4]. Thus, the use of plant extracts and their phytoconstituents as active ingredients is a modern “pro-ecological” approach [5,6]. The increasing demand of these products can be due to their reduced side effects, their broad spectrum of action combined with a high efficacy, and their generally low prices [7,8].

Plants are rich in several classes of bioactive compounds, being one of the most plentiful sources of new ingredients responsible for treating many diseases [9,10]. In addition, plants are also sources of natural moisturizers, flavorings, and pigments, which make them very interesting for skin cosmetic applications [5]. Finally, plant extracts are generally considered safe and fulfill the requirements of the regulatory authorities [10,11].

Among the compounds present in plant extracts, phenolics have gained special attention as active ingredients [12,13], mainly because they stand out as anti-inflammatory, antimicrobial, and antioxidant agents [14,15]. These properties make them ideal preventive and healing molecules for skin disorders, being applied in cosmetology and dermatology [16]. The noticeable antioxidant activity of phenolics is also partially responsible for their anti-aging effects, which are possibly due to their ability to reduce collagen degradation and in provide UV protection [16]. Therefore, the use of natural phenolic-rich extracts with high antioxidant capacity have been investigated and encouraged for the replacement of synthetic antioxidants in skin products [12].

Natural products extracted from plants of Malvaceae family are used worldwide, and the genus *Hibiscus* has gained great attention for the multiple pharmacological activities of their extracts and for their high phenolics abundance [17,18,19]. *Hibiscus* spp. contains about 240 species of annual or perennial flowering herbs, shrubs, or trees, which are distributed in different regions of the world [20]. *Hibiscus* extracts have been applied in traditional medicine as emollients for the treatment of many skin disorders and burns [19,21]. Based on these literature data, extracts from *Hibiscus* sp. plants might be interesting active ingredients for skin cosmetic formulations, protecting the cells from oxidative stress, collagen degradation, and against harmful effects of UV radiation.

Although the genus *Hibiscus* comprises many species, less than 10% of them have been investigated so far [17]. *Hibiscus roseus* Thore (syn. *H. palustris* L., *H. moscheutos* subsp. *palustris* (L.) R. T. Clausen.) is anherbaceous perennial species naturalized in Italy [22,23]. The identification and description of *H. roseus* is still under debate [20,23]. According to the literature, the species *H. moscheutos* subsp. *palustris* has been introduced very early in Europe whereas in France, it had been described as a new species, *H. roseus*, by Thore in 1807 [23]. This species has not been characterized for its phenolic composition and studied for its biological activities, which makes it a potential unrevealed source of bioactive compounds for skin-care products.

The folk use in skin treatments and the broad spectrum of bioactivities of *Hibiscus* species justify the importance of new studies focused on this plant genus [17]. Therefore, the aim of this study was to characterize the phenolic composition and to evaluate the antioxidant capacity, sun protection, and collagenase inhibition activity of the ethanolic extracts of leaves and flowers of *H. roseus*. Our results present for the first time the phenolic composition and anti-aging-related bioactivities of *H. roseus*, indicating the potential of this under-investigated species in the medicinal and cosmetic applications as an antioxidant and anti-aging additive.

## 2. Results and Discussion

### 2.1. Phenolic Characterization and Quantification

A target analysis, based on LC-MS/MS-MRM (liquid chromatography coupled with tandem mass spectrometry working in multiple reaction monitoring mode), was conducted to tentatively identify the phenolic compounds present in ethanolic extracts of *H. roseus*, since the phenolic composition of this species has not been still reported in the literature. Nineteen phenolic compounds previously described in Hibiscus genus were used as standards (Supplementary Table S1) to develop the MRM method, with the selection of the best transitions being designed by the optimization of the instrumental parameters and by literature data [24].

**Figure 1:**
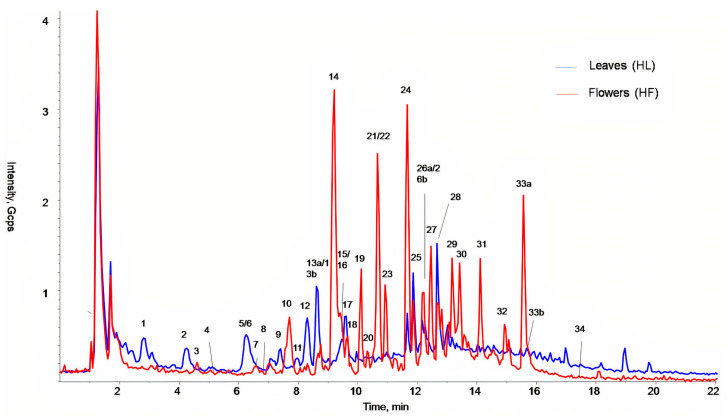
HPLC-MS (Scan 100–1000 Da, negative ions) chromatogram of *Hibiscus roseus* ethanolic extract of leaves (blue line) and flowers (red line).

The main classes of compounds detected in *H. roseus* leaf and flower extracts were chlorogenic, *p*-coumaric, and *trans*-ferulic acids derivatives and flavonoid derivatives (Figure 1, Table 1), similarly to previous phytochemical characterization of other *Hibiscus* species [25,26,27,28,29]. Although the phenolic profile was quite similar, some qualitative differences were observed between flowers (HF) and leaves (HL) (Figure 1 and Table 1). While leaves showed richness in *p*-coumaric acid derivatives (Figure 1, blue line, peaks with Rt from 2 to 9 min), flowers were especially rich in flavonoid derivatives such as catechins, dihydrochalcones, and anthocyanins (Figure 1, red line, Rt > 9.3 min, Table 1).

Thirteen of the nineteen target phenolic compounds were authentically identified in the extracts analyzed by LC-MS/MS in the MRM mode (Table 1). The MRM is a powerful way for the simultaneous determination of several components, based on the mass-to-charge ratio (*m*/*z*) of the molecular ion ([M−H]^−^) and its corresponding daughter ion. It allows the enhancement of selectivity and sensitivity of LC-MS/MS analyses [30]. This methodology is very reliable and suitable for analyses of plant extracts and other complex mixtures leading to the highest specificity, excellent sensitivity, and an extreme multiplexing capacity thanks to the possibility of distinguishing compounds having the same parent ions but different fragments [31,32]. Using this method, we have obtained a significant reduction of chromatographic runs, a higher specificity and accuracy provided by a good separation of compounds detected with the same transitions, while avoiding a loss of sensitivity in the case of different co-eluting compounds or for compounds present in very low concentration [24,33,34].

Among the 13 phenolic compounds identified utilizing authentic standards (Supplementary Table S1), ten were exclusively present in flower extracts (Figure 1 red line, Table 1 HF): catechin and epicatechin (peaks 16b and 23), chlorogenic acid (peak 18), peonidin-3-*O*-glucoside and oenin (peaks 21 and 22), trans-ferulic acid (peak 27), three kaempferol glycoside derivatives (kaempferol-3-*O*-rutinoside, kaempferol-7-*O*-glucoside, and kaempferol-3-*O*-glucoside; peaks 30, 31b, and 32), and tiliroside (peak 34). Additionally, phloridzin (peak 33b) was detected only in leaf extracts (Figure 1 blue line, Table 1 HL), while rutin and quercetin-3-*O*-glucoside (peaks 26b and 28a) were identified in both type of extracts (Figure 1, Table 1 HF/HL). Similar quercetin derivatives, such as quercetin-3-*O*-sambubioside and isoquercitrin, were previously observed in *H. sabdariffa* [26,27,29,35,36] and in *H. rosa-sinensis* extracts [18]. Some of these glycosides could correspond to the quercetin derivatives that we detected in *H. roseus*. In addition, tiliroside has been also previously detected in phenolic extracts of *H. sabdariffa* flowers [37,38]. Oenin (malvidin-3-*O*-glucoside) and peonidin-3-*O*-glucoside, the two anthocyanins here identified in *H. roseus* flowers for the first time, were different from those previously described in *H. sabdariffa* flowers, delphinidin 3-sambubioside, delphinidin-3-glucoside, and cyanidin-3-*O*-sambubioside [27,35,39,40]. However, it is important to mention that the most studied part of the flowers of *H. sabdariffa* is the calyx (sepals), not the petals as investigated here for *H. roseus*.

In addition to the compounds identified and confirmed by the authentic target standards, another 27 compounds were putatively identified in *H. roseus* leaf and flower extracts based on their MRM (*m*/*z*) and their daughter ions, thus considering the fragmentation products obtained from the precursor (Supplementary Table S1). In particular, the presence of *p*-coumaric, *trans*-ferulic, and chlorogenic acid derivatives, and quercetin derivatives, as well as phloretin and phloridzin derivatives were found in both extracts (Table 1).

The quantification of the phenolics identified in these extracts was performed by HPLC-DAD analysis (high-performance liquid chromatography coupled to diode array detection; Table 2). The content of hydroxycinnamic acid derivatives (THC) was higher in leaves than in flowers, while greater amounts of flavonoids (TFC) were found in flowers than in leaves (*p* < 0.001, Table 2). Catechin derivatives (TCD), dihydrochalcones (TDC), and anthocyanins (TAC) were quantified only in flower extracts (Table 2, *p* < 0.001). Therefore, flowers represent a greater source of phenolics compared to leaves (TPC, *p* = 0.002, Table 2). Similarly to *H. sabdariffa* extracts, the major classes of compounds found in *H. roseus* leaves were chlorogenic and *p*-coumaric acid derivatives as well as caffeoylquinic and *p*-coumaroylquinic acids [26,40,41]. In addition, anthocyanins were exclusively reported in *Hibiscus* spp. flowers and calyxes, together with catechins [19,27,28,35].

By contrast, ferulic acid and its derivatives were less reported as constituents of *Hibiscus* spp. extracts, but they may be of great importance for their biological activities [6,42,43,44]. Indeed, ferulic acid derivatives obtained from different *Hibiscus* species showed important pharmacological properties such as antiviral and angiotensin-converting enzyme inhibitory activities [43,44]. In addition, ferulic acid was described as an active molecule in *H. mutabilis*, *H. taiwanensis* extracts [45,46], and in *H. sabdariffa* calyx extracts [28,38].

Regarding the potential cosmetic applications, it has been proven that ferulic acid inhibits melanin formation [6,42], while *p*-coumaric acid derivatives possess depigmentation [47,48], anti-inflammatory, and tyrosinase inhibition activities [47,49]. In addition, many investigations highlight additional roles of flavonols and anthocyanins, which may act as skin protective compounds, in particular inhibiting melanogenesis [50,51] and through their action as anti-aging compounds and preventing melanoma [52,53]. In addition, the potential applications of *H. roseus* leaf extracts for skin disorders could be also enhanced by the presence of phloridzin, which has shown to decrease the expression of UVB-induced pro-inflammatory cytokines in UV-exposed skin [54].

### 2.2. Antioxidant Activity Assays

Nowadays, it is widely demonstrated that the accumulation of reactive oxygen species (ROS) is responsible for skin-aging processes, leading to dryness, losses of subcutaneous tissue, and wrinkles formation [55,56]. Therefore, finding natural potential antioxidant compounds that can be applied in skin-care products is very important for cosmetic industries.

Our results showed that *H. roseus* leaf extracts had lower antioxidant activity (expressed as EC_50_ values) than flowers (Table 3). Indeed, the antioxidant activities of flowers extracts were at least two times greater than those of the leaf extracts in both assays (Table 3). These results agree with the phenolic composition and content of these extracts (Figure 1, Table 1 and Table 2), since HF extracts were richer in phenolic compounds (Table 2). Indeed, the correlation analysis between the EC_50_ values and the content of the different classes of phenolics showed to be significant and negative for all the compounds except for THC. As such, higher amounts of flavonoids, catechins, anthocyanins, dihydrochalcones, and total phenolic content contribute to greater antioxidant capacities (lower values of EC_50_—Table 4).

Among flavonoids, quercetin and its derivatives are the most well-established antioxidant and free radical scavengers, also acting as effective inhibitors of oxidases and lipoxygenases [57]. Moreover, dihydrochalcones, such as phloretin, have also been described as potent antioxidants in 2,2-diphenyl-1-picrylhydrazyl (DPPH)-scavenging and OH-scavenging assays [58]. In addition, anthocyanins isolated from *Hibiscus* extracts showed to be major antioxidant compounds in human cells [59].

Extracts of different parts of *Hibiscus* species have shown high antioxidant capacity [18,21,27,35,40]. Fractions of ethanolic extracts of *H. sabdariffa* showed very low EC_50_ values in antioxidant assays, which were correlated to the high content in protocatechuic acid [21,59], chlorogenic acid, flavonoids, and anthocyanins [24,60]. In addition, a study on *H. esculentus* showed the in vitro antioxidant potential of quercetin derivatives and catechins present in its extracts [61]. Finally, in *H. acetosella*, the antioxidant activity was strongly correlated with the anthocyanins content [62].

The results of our study on *H. roseus* ethanolic extracts showed an antioxidant activity that was a hundred times higher than those reported for aqueous extracts of *H. sabdariffa* calyx, for which the EC_50_ was near to 45 mg mL^−1^ in a similar DPPH in vitro model [56]. However, in distinction to our findings, the total flavonoid content and the antioxidant capacity of *H. sabdariffa* leaf extracts were higher than those of flowers [63,64].

### 2.3. In Vitro Sun Protection Factor (SPF)

Ultraviolet radiation is one of the most harmful environmental factors influencing the health and physiology of the skin, being an important extrinsic skin-aging cause [65,66]. Constant exposure to ultraviolet radiation increases the risk of pigmentation disorders and skin photoaging [67]. This is mostly due to the increase in ROS levels, which leads to the stimulation of collagenase production and results in considerable damage to skin cellular functions [56]. Therefore, UV-protecting ingredients, including those present in plant extracts, are widely applied in cosmetics to avoid the penetration of ultraviolet radiation in the skin but also prevent ROS production by acting as antioxidants [56,68].

A simple method to verify the efficacy of different natural components as UV filters is the sun protection factor (SPF) assay, which is a rapid and reliable in vitro method based on the screening of the absorbance within UV-B spectral region (between 290 and 320 nm), being useful in an early phase of selection of photoprotection active ingredients [69].

The high phenolic content and antioxidant activity of *H. roseus* extracts suggest that they may have also an UV absorbing activity. Both leaf and flower extracts of *H. roseus* at 0.1 mg mL^−1^ showed comparable SPF results (*p* > 0.05): 2.6 ± 0.15 for HL and 2.4 ± 0.19 for HF. These results are promising, since a standard sunscreen formulation containing 8% homosalate (a widely applied chemical sunscreen) showed an SPF value of 4 [69,70]. The results found here for *H. roseus* were similar to those found for other plant species [68,69,71,72] and are important considering the low concentration of the extracts used to test this effect.

Extracts of *H. rosa-sinensis* have already shown positive effects against the ultraviolet radiation damages in mouse skin by means of antioxidant protection [73]. Natural products exhibiting SPF together with high antioxidant capacities and the inhibition of collagenase and elastase are important candidates to be used to protect the skin from photodamage and to prevent the appearance of wrinkles [66,71]. In fact, the association between approved traditional sunfilters and those derivatives of natural sources represents a trend in the cosmetic industry, since consumers perceive these products as safer, due to the side effects of synthetic UVfilters [72].

The higher content of total phenolic compounds of HF extracts (TPC; Table 2) could indicate their higher UV absorbing activity. However, both leaf and flower extracts showed very similar results, indicating that more than the total content of phenolics, the phenolic profile of the extracts would be related to the protection against UV. In particular, the higher content of hydroxycinnamic acid derivatives in HL (Table 2) may contribute to increase their SPF value, since these compounds have an UV absorption around 300–320 nm [74], which is thus centered in UV-B region. Conversely, flavonoids and anthocyanins, mostly present in flowers extracts, have a broader spectrum of absorbance in which at least two bands are present, with the major band in or near to the visible range, around 350 nm for flavonols and 505–550 nm for anthocyanins [53,69]. Indeed, hydroxycinnamic acid derivatives are produced by plants especially for their protection against UV radiation [75]. Therefore, these hydroxycinnamic acid derivatives could greatly contribute to the absorption of UV-B by human skin [6]. However, considering the presence of anthocyanins and flavonoids that cover a boarder range of wavelengths absorption, also including the UV-A and visible regions, *H. roseus* flowers extracts might be promising for further analysis and the development of sunblock cosmetic products. In addition, the higher antioxidant activity observed for HF (Table 3) could enhance the sun-protection effects in possible further formulations [69].

### 2.4. Collagenase Inhibition Activity

**Figure 2:**
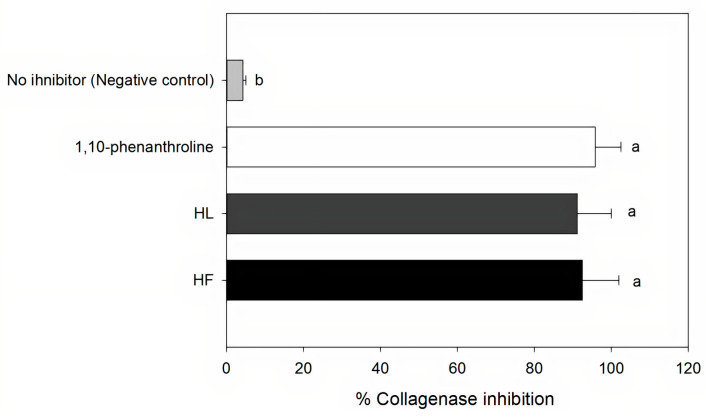
Collagenase inhibitory activity (in percentage) of extracts of *Hibiscus roseus* flowers (HF) and leaves (HL) at 0.25 mg mL^−1^, and controls (negative control—no inhibitor—and 1,10-pheanthroline 1M—positive control). Mean values and standard deviation (*n* = 3), different letters indicate significant differences among the samples (*p* ≤ 0.05).

Both *H. roseus* extracts showed high collagenase inhibitory activity (>80%) at 0.25 mg mL^−1^, which is comparable to that of the synthetic inhibitor 1,10-phenanthrolineat 1M (Figure 2). The IC_50_ value of both extracts were very similar (*p* > 0.05), IC_50flower extracts_ = 0.14 ± 0.02 mg mL^−1^ and IC_50leaf extracts_ = 0.13 ± 0.01 mg mL^−1^, despite their differences in phenolic composition and content (Table 1 and Table 2). This could be due to the synergistic interactions between the phenolics and collagenase, which could play an important role in the inhibition mechanism. In addition, other compounds possibly present in the *H. roseus* extracts and not analyzed here might take part in the anti-collagenase activity, including vitamin E and ascorbic acid [71,76,77].

Moreover, the two tested standard compounds, chlorogenic acid and quercetin, whose derivatives are present in *H. roseus* leaf and flower extracts (Table 1), exhibited very high collagenase inhibition, with IC_50_ values of 5.8 ± 0.5 and 5.6 ± 0.7 μg mL^−1^, respectively. Therefore, these compounds might be responsible for the observed anti-collagenase activity. It is relevant to notice that different classes of phenolics, which are also present in our plant extracts, have already shown anti-aging activity via the inhibition of collagen degradation and contributing to skin humidification [78]. For example, ferulic acid and its derivatives have been proven to moisturize the skin and stimulate the synthesis of collagen fibers, being used in cosmetics such as anti-wrinkle creams [6]. Furthermore, flavonols, in particular quercetin derivatives, are strong inhibitorsof the collagenase enzyme [79].

Our results show the promising effect of *H. roseus* extracts against the degradation of collagen, which is one of the greatest proteins responsible for losses in skin elasticity and integrity and in the formation of wrinkles [80,81]. The collagenase enzyme inhibits the retention of skin elasticity and tensile strength [82]. Indeed, different studies have shown the importance of natural antioxidants due to their efficacy in delaying the premature aging through the inhibition of collagenase activity [78,83].

Previous studies evaluating the effects of *Hibiscus* species in the stimulation of collagen production and in the inhibition of collagenase activity have been conducted [56,84,85]. The collagenase activity inhibition of *H. sabdariffa* aqueous extracts has been recently described in literature [56]. Similar to our findings, the authors did not observe effects of collagenase inhibition at low concentrations of the extracts but only at significant high concentrations [56]. In a different study, the IC_50_ value in collagenase inhibition of *H. sabdariffa* ethanolic extracts was 0.75 ± 0.04 mg mL^−1^ [65], which is an activity that is almost six times lower than those described here for *H. roseus*.

## 3. Materials and Methods

### 3.1. Plant Material

Ten *Hibiscus roseus* Thore plants, bought from a commercial nursery in Florence (Italy), were planted in 10-liter pots filled with sandy soil (sand/peat, 60:40, *v*/*v*) and maintained in the greenhouse of the Department of Agriculture, Food, Environment and Forestry (DAGRI)—University of Florence (UNIFI), Sesto Fiorentino (Italy, 43°49′ N, 11°37′ E). The plants were cultivated in the greenhouse from January to July 2019, under manual irrigation at the pot water capacity. From these ten different plants, two-pooled leaves and flowers were collected at the end of July during the flowering period and immediately stored at −80 °C until the extraction.

### 3.2. Ultrasound-Assisted Extraction

Lyophilized samples (900 mg) of *H. roseus* flowers (HF) and leaves (HL) were ground in liquid nitrogen and extracted with 3 × 15.0 mL ethanol 75% (pH2.5 adjusted with HCOOH) by an ultrasound-assisted extraction (UAE). The UAE was conducted in an ultrasonic bath (BioClass^®^ CP104) using a constant frequency of 39 kHz and an input power of 100 W, during 30 min, at 5 °C. After centrifugation (5 min, 9000 rpm, 5 °C; ALC^®^ 4239R, Milan, Italy), the supernatants were partitioned with 3 × 15 mL of n-hexane to remove lipophilic compounds that could interfere with the analysis. The ethanolic phase was reduced to dryness, weighted on a digital analytical balance (Precisa^®^ 125A), and the residue was resuspended with methanol/water acidified solution (1:1 *v*/*v*, pH2.5 adjusted with HCOOH). The extraction process was carried out in triplicate.

### 3.3. LC-MS Analysis: Phenolic Profile of the Extracts

The LC-MS analysis was conducted using an ABSciex API 3000 triple quadrupole mass spectrometer (AB Sciex LLC, Framingham, MA, USA) coupled with an Agilent 1100 HPLC system with binary pump and autosampler (Agilent Technologies, Inc., Santa Clara, CA, USA). Acquisition and data reduction were performed using Analyst 1.6.2 software (AB Sciex LLC, Framingham, MA, USA).

The HPLC separation was carried out on an Agilent Phenyl Column (3 × 100 mm; 2.7 μm), and the eluents were (A) acidified water (at pH2.5 adjusted with HCOOH) and (B) acetonitrile/water (90/10, at pH2.5 adjusted with HCOOH). A gradient solvent system was used as follows: 0–3 min, 5% B; 3–18 min, 5–40% B; 18–28 min, 40% B; 28–38 min, 40–80% B; 38–43 min, 80% B, 43–45 min, 80–5% B, at a flow rate of 0.4mLmin^−1^.The MS analysis was carried out under the following experimental conditions: Atmospheric Pressure Chemical Ionization (APCI) using the heated nebulizer interface; Needle Current (NC), −5 µA; Nebulizer Gas (air), 10 (arbitrary units); Auxiliary Gas (air), 3 L min^−1^; Auxiliary Gas Temperature (TEM), 550°C; Curtain Gas (CUR, nitrogen), 6 (arbitrary units); Collision Gas (CAD, Nitrogen), 9 (arbitrary units, corresponding to 2.6 × 10^−5^ Torr collision cell pressure).

The identification of the different phenolic components was performed using a targeted approach, using a multiple reaction monitoring (MRM) method, optimized with standards for 19 target compounds (chosen based on previous studies of polyphenolic composition of *Hibiscus* spp. [25,35]): two flavan-3-ols (catechin and epicatechin), seven flavonols (quercetagetin-7-*O*-glucoside, rutin, quercetin-3-*O*-glucoside, kaempferol-3-*O*-rutinoside, kaempferol-7-*O*-glucoside, kaempferol-3-*O*-glucoside, and quercetin), one cinnamate ester (chlorogenic acid), two hydroxycinnamic acids (*p*-coumaric and trans-ferulic acids), two dihydrochalcones (phloridzin and phloretin), one oxyflavone (tiliroside), and four anthocyanins (myrtillin, kuromanin, peonidin-3-*O*-glucoside, and oenin). The retention time and the relative MRM transitions (quantifier and qualifier) were reported in the Supplementary Table S1. Moreover, additional tentative identifications have been suggested using an untargeted approach, scanning the quadrupole from *m*/*z* 100 to 1000 Da.

### 3.4. HPLC-DAD Analysis: Quantification of Phenolics

HPLC-DAD analysis was performed to quantify the different classes of phenolics (hydroxycinnamic acid derivatives, catechins, dihydrochalcones, flavonoids, and anthocyanins) in the extracts. Aliquots of the samples (15 μL) were injected into a Perkin^®^ Elmer Flexar liquid chromatograph equipped with a quaternary 200Q/410 pump and an LC 200 diode array detector (DAD) (all from Perkin Elmer^®^, Bradford, CT, USA). The chromatographic conditions were the same as those used for HPLC-MS/MS analyses (Section 3.3).

The chromatograms were acquired at 280, 330, 350, and at 520 nm (for the quantification of anthocyanins). The identification and quantification of the phenolic compounds were carried out based on the retention time, UV spectral characteristics, and comparison with standards, as well as based on literature data [25,35] and in the previous LC-MS analysis. Five-point calibration curves with different standards (chlorogenic acid, *p*-coumaric, rutin, epicatechin, naringin, and peonidin-3-*O*-glucoside, all from Sigma–Aldrich^®^–Merck^®^KGaA, Darmstadt, Germany) were used to quantify the different polyphenols detected and identified in the extracts. If a commercial standard was not available, the quantification was performed using the calibration curve of standards from the same phenolic class, giving an estimated content. The linearity of the curves was determined by the coefficient of determination (R^2^), being higher than 0.99 for all the standards.

All the extracts were analyzed in triplicate and the quantitative results of the phenolics were given in mg g^−1^ of dry weight (mg g^−1^ DW), being expressed as total hydroxycinnamic acid derivatives content (THC), total flavonoids content (TFC), total catechin derivatives content (TCD), total dihydrochalcones content (TDC), total anthocyanins content (TAC), and total phenolic content (TPC), which were estimated as the sum of the individual identified compounds belonging to each class.

### 3.5. Antioxidant Activity Assays

The antioxidant activity assay was performed using two different methods: DPPH (2,2-diphenyl-1-picrylhydrazyl) and the Hydroxyl Radical (OH)-Scavenging (HRS) assays.

The method of Khandi and Charles [86] was applied for the DPPH assay. Briefly, diluted samples of the extracts (0.5 mL) were added to 0.5 mL of DPPH solution (0.1 mM in methanol; Sigma-Aldrich^®^, St. Louis, MI, USA), and the mixture was left to react at room temperature for 40 min in the dark. This time (40 min) was defined based on the kinetic analyses results of each extract and the standards chlorogenic acid and rutin. After the reaction time, the absorbance was measured at 518 nm using a PerkinElmer^®^ Lambda 25UV/VIS spectrophotometer. The absorbencies of blank (0.5 mL methanol and 0.5 mL samples) and of the negative control (0.5 mL methanol and 0.5 mL DPPH solution) were also evaluated. All the analyses were conducted in triplicate. The percentage of antioxidant activity was calculated as follows (1).

AA% = 100 − {[(ABS_sample_ − ABS_blank_) × 100]/ABS_negative control_} (1)

The Hydroxyl Radical-Scavenging (HRS) assay was performed following the method of Smirnoff and Cumbes [87], with some modifications [88]. Different concentrations of the extracts were left to react with FeSO_4_ (1.5 mM), hydrogen peroxide (6 mM), and sodium salicylate (20 mM), at 37 °C for 1 h. Afterwards, the absorbance was measured at 562 nm.

The EC_50_ values (concentration of the extract sufficient to obtain 50% of the total antioxidant activity) from both methods were calculated with the Microsoft Excel^®^ software.

### 3.6. In Vitro Sun Protection Factor (SPF) Assay

The SPF analysis was determined according to Gaweł-Beben et al. [68] by measuring the absorbance of the extracts (at 0.1 mg mL^−1^ in methanol: water 50%) within the wavelength range from 290 to 320 nm, with intervals of 5 nm and using 50% (*v*/*v*) methanol/water solution as blank. The absorbencies were measured using a PerkinElmer^®^ Lambda 25 UV/VIS spectrophotometer.

Equation (2) obtained by Mansur et al. [70] was applied to calculate the SPF, using the *EE* × *I* values determined by Sayre et al. [89] (Table 5).

(2) $$SPF=CF\times \sum_{290}^{320}EE\left(\lambda \right)\times I\left(\lambda \right)\times Abs\left(\lambda \right)$$

where *EE* (*λ*)—erythemal effect spectrum; *I* (*λ*)—solar intensity spectrum; *Abs* (*λ*)—absorbance of the sample; *CF*—correction factor (=10).

### 3.7. Collagenase Activity Inhibitory Assay

The collagenase inhibitory assay was performed similarly to those described by Roda et al. [83], using a Collagenase Activity Assay Kit (Sigma-Aldrich^®^). This kit measures the collagenase activity with a synthetic peptide (i.e., FALGPA; N-(3-[2-Furyl]acryloyl)-Leu-Gly-Pro-Ala) that mimics the collagen structure. According to the manufacturer instructions, aliquots (2 μL) of the extracts at concentrations ranging from 0.1 to 1.0 mg mL^−1^ were spiked with collagenase (0.35 U/mL, 10 μL) and assay buffer (88 μL) in 96-well plates. An enzyme control (10 μL of collagenase + 90 μL of buffer), an inhibitor control (2 μL of 1,10-phenanthroline 1M + 10 μL of collagenase + 88 μL of buffer), and a blank (100 μL of buffer) were also prepared. Aliquots (2 μL) of two standards (chlorogenic acid and quercetin, both from Sigma-Aldrich^®^) at concentrations ranging from 0.5 to 10.0 μg mL^−1^ were also evaluated. The reaction was started by adding FALGPA-buffer solution to each well (100 μL), and the absorbencies were immediately measured at 345 nm for 20 min for 3 min each, using a SpectraMax^®^ reader. The reaction time was defined after a previous kinetic test. The collagenase inhibition was calculated as follows (3):

(3) $$Collagenase\;activity\;\left(\frac{U}{mL}\right)=\frac{\left(\frac{-\Delta A345nm}{\Delta T}extract-\frac{-\Delta A345nm}{\Delta T}blank\right)\times RV\times DF}{EC\times V}$$

where Δ*A*_345*nm*_ is the absorbance difference between the beginning and the end of the acquisition; Δ*T* is the time difference between the beginning and the end of the acquisition, RV is the reaction volume (0.2 mL); DF is the dilution factor; EC is the extinction coefficient of collagenase substrate (0.53 mM), and V is the enzyme volume (mL). All the analyses were performed in triplicate.

For both extracts (HL and HF) and for the standards, the percentage of collagenase inhibition was determined (4). Similarly to the antioxidant capacity, the results were reported as the extract concentration providing 50% of enzyme inhibitory activity (IC_50_).

Inhibition (%) = [(Activity enzyme − Activity inhibitor)/Activity enzyme)] × 100(4)

### 3.8. Statistical Analysis

The results of the content of phenolics, antioxidant capacities, SPF, and collagenase inhibition activity of the extracts were expressed as mean ± standard deviation (SD) (*n* = 3). A Student’st-test was used to compare the results (flowers vs leaves samples). A correlation analysis was performed between the antioxidant activity (DPPH assay) and the respective content of classes of phenolics (HPLC-DAD quantification) using the Pearson correlation test. All the statistical analyses were performed using SigmaPlot^®^ Systat^®^ software (version 12.5) and the differences considered significant when *p* ≤ 0.05.

## 4. Conclusions

Secondary metabolites are potential active ingredients for cosmetic new formulations. Among these, phenolic compounds extracted from plants may have great antioxidant and anti-aging properties, being effective in the inhibition of dermal enzymes (e.g., collagenase) and in UV absorption. Therefore, under-investigated plant extracts, such as those of *H. roseus*, can represent unrevealed sources of bioactive molecules.

We demonstrated that the leaves and flowers of *H. roseus* are rich in hydroxycinnamic acid derivatives and flavonoids, with flowers having greater amounts of kaempferol glycosides, catechins, dihydrochalcones, and anthocyanins, all of these compounds not described yet in the literature for this species. The great antioxidant capacity, especially of flowers extracts, together with the sun-protection and anti-collagenase activity of both leaf and flowers extracts, point out the promising utilization of this poorly investigated species in skin-care applications. In conclusion, our results showed the potential of *H. roseus* flowers and leaves as sources of phenolics as well as the activity of their extracts as anti-aging agents that might be used as ingredients for functional cosmetic products.

## Figures and Tables

**Table 1:** Putative identification of the main phenolic compounds found in leaf (HL) and flowers (HF) extracts of *Hibiscus roseus* by LC-MS/MS-MRM. Compounds numbers correspond to those indicated in Figure 1.

Peak	Retention Time (min)	Extract	Putative Identification	Authentic Standard Identification
1	2.9	HL/HF	chlorogenic acid derivative	No
2	4.4	HL	*p*-coumaric acid derivative	No
3	4.7	HF	*p*-coumaric acid derivative	No
4	5.7	HF	*p*-coumaric acid derivative	No
5	6.3	HL	*p*-coumaric acid derivative	No
6	6.4	HL/HF	*trans*-ferulic acid derivative	No
7	6.5	HF	chlorogenic acid derivative	No
8	6.7	HF	*p*-coumaric acid derivative	No
9	7.5	HL	*p*-coumaric acid derivative	No
10	7.8	HF	*p*-coumaric acid derivative	No
11	8.0	HL	*trans*-ferulic acid derivative	No
12	8.4	HL	*p*-coumaric acid derivative	No
13a	8.7	HF/HL	*p*-coumaric acid derivative	No
13b	8.7	HF/HL	chlorogenic acid derivative	No
14	9.0	HF	*trans*-ferulic acid derivative	No
15	9.2	HL	chlorogenic acid derivative	No
16a	9.3	HF	catechin	Yes
16b	9.3	HF	quercetin derivative	No
17	9.6	HF/HL	*trans*-ferulic acid derivative	No
18	9.7	HF	chlorogenic acid	Yes
19	10.3	HF	*trans*-ferulic acid derivative	No
20	10.7	HF/HL	chlorogenic acid derivative	No
21	10.8	HF	peonidin-3-*O*-glucoside	Yes
22	10.9	HF	oenin	Yes
23	11.0	HF	epicatechin	Yes
24	11.7	HF/HL	quercetin derivative	No
25	12.2	HF/HL	kaempferol derivative	No
26a	12.7	HF/HL	quercetin derivative	No
26b	12.7	HF/HL	rutin	Yes
27	12.8	HF	*trans*-ferulic acid	Yes
28a	13.2	HF/HL	quercetin3-*O*-glucoside	Yes
28b	13.2	HL	phloridzin derivative	No
29	13.4	HF	quercetin derivative	No
30	13.6	HF	kaempferol-3-*O*-rutinoside	Yes
31a	14.1	HF	phloretin derivative	No
31b	14.1	HF	kaempferol-7-*O*-glucoside	Yes
32	14.7	HF	kaempferol-3-*O*-glucoside	Yes
33a	15.6	HF	phloretin derivative	No
33b	15.6	HL	phloridzin	Yes
34	17.4	HF	tiliroside	Yes

**Table 2:** Quantification of phenolics (mg g^−1^ dry weight, DW) in *Hibiscus roseus* leaf (HL) and flower (HF) extracts. TFC: total flavonoid content; THC: total hydroxycinnamic acid derivatives content; TCD: total catechin derivatives content; TDC: total dihydrochalcones content; TAC: total anthocyanins content; TPC: total phenolic content.

*H. roseus* Extracts	THC	TFC	TCD	TDC	TAC	TPC
Leaves (HL)	5.08 ± 0.48 ***	3.78 ± 0.22	nd	nd	nd	8.86 ± 0.70
Flowers (HF)	1.31 ± 0.13	6.26 ± 0.28 ***	1.86 ± 0.04 ***	2.18 ± 0.06 ***	0.35 ± 0.03 ***	11.96 ± 0.48 **

Results given in mean ± SD (*n* = 3), nd: not detected by high-performance liquid chromatography coupled to diode array detection (HPLC-DAD) analysis, or because they are not present in the extracts or due to their low quantity; *** *p* < 0.001; ** *p* < 0.01, comparison between flowers and leaf extracts.

**Table 3:** Antioxidant activity (in terms of EC_50_) of extracts of *Hibiscus roseus* leaves and flowers.

	EC_50_ Values (mg mL^−1^)	
*H. roseus* Extracts	DPPH Assay	HRS Assay
Leaves (HL)	0.38 ± 0.05	2.44 ± 0.23
Flowers (HF)	0.24 ± 0.009 **	0.88 ± 0.06 ***

EC_50_ values (in mg mL^−1^) given in mean ± SD (*n* = 3); *** *p* < 0.001; ** *p* < 0.01, comparison between flowers and leaves extracts.

**Table 4:** Pearson correlation analysis between the antioxidant capacity (EC_50_ values) and the phenolic content of *Hibiscus roseus* leaves and flowers extracts.

Phenolic Content	Pearson Coefficient—*r* (EC_50_ Values)	*p*-Value
THC	0.92	0.009 **
TFC	−0.87	0.02 *
TCD	−0.92	0.01 **
TDC	−0.91	0.01 **
TAC	−0.92	0.01 **
TPC	−0.94	0.004 **

Asterisks show significant correlations among the parameters (* *p* ≤ 0.05; ** *p* ≤ 0.01).

**Table 5:** Normalized product function used in the calculation of sun protection factor (SPF).

Wavelenght (λ, nm)	*EE* × *I* (Normalized)
290	0.0150
295	0.0817
300	0.2874
305	0.3278
310	0.1864
315	0.0839
320	0.0180
Total	1.0002

*EE*—erythremal effect spectrum, *I*—solar intensity spectrum.

## Data Availability

The data presented in this study are available on request from the corresponding author. The data are not publicly available due to the further potential uses in patents and in products development.

## Supplementary Materials

The following are available online at https://www.mdpi.com/2223-7747/10/3/522/s1, Table S1: Retention time and transitions of the selected standards analyzed by LC-MS/MS-MRM (liquid chromatography coupled with tandem mass spectrometry working in multiple reaction monitoring mode).
