# Supplementary material for: Phenolic Compounds from Leaves and Flowers of Hibiscus roseus: Potential Skin Cosmetic Applications of an Under-Investigated Species
Source: Plants (Basel). 2021 Mar 10;10(3):522. doi: 10.3390/plants10030522 (PMC8000889; doi:10.3390/plants10030522)
Supplement: Supplementary file 1 [file plants-10-00522-s001.pdf]

Supplementary Table S1: Retention time and transitions of the selected standards analyzed by LC-MS/MS-MRM (liquid chromatography coupled with tandem mass spectrometry working in multiple reaction monitoring mode).

Component (Neg. Ions)	Ret. Time (min)	Quantifier Transition	Qualifier Transition	Quant/Qual Area Ratio
Catechin	9.17	289→245	289→203	2.4
Chlorogenic Acid	9.67	353→191	353→85	25.1
Epicatechin	10.85	289→245	289→203	3.1
Quercetagetin 7-*O*-Glucoside	12.05	479→317	479→166	11.7
p-Coumaric Acid	11.68	163→119	163→93	17.8
Rutin	12.70	609→300	609→271	6.1
Trans-ferulic Acid	12.75	193→134	193→149	3.4
Quercetin 3-*O*-Glucoside	13.15	463→300	463→271	5.4
Kaempferol 3-*O*-rutinoside	13.61	593→285	593→255	5.2
Kaempferol 7-*O*-glucoside	14.13	447→285	447→151	17.7
Kaempferol 3-*O*-glucoside	14.75	447→285	447→151	111.2
Phloridzin	15.67	475→273	475→167	3.2
Tiliroside	17.42	593→285	593→255	5.4
Quercetin	17.53	301→151	301→179	3.5
Phloretin	19.48	273→167	273→123	3.8
Component (Pos. Ions)	Ret. Time (min)	Quantifier Transition	Qualifier Transition	Quant/Qual Area Ratio
Myrtillin	8.96	465→303	465→229	84.3
Kuromanin	9.91	449→287	449→213	154.2
Peonidin-3-*O*-glucoside	10.85	463→301	463→286	5.1
Oenin	10.88	493→331	493→315	5.7
